# Exosomes Secreted by Umbilical Cord Blood-Derived Mesenchymal Stem Cell Attenuate Diabetes in Mice

**DOI:** 10.1155/2021/9534574

**Published:** 2021-12-10

**Authors:** Rajni Sharma, Manju Kumari, Suman Mishra, Dharmendra K. Chaudhary, Alok Kumar, Batia Avni, Swasti Tiwari

**Affiliations:** ^1^Department of Molecular Medicine & Biotechnology, Sanjay Gandhi Post Graduate Institute of Medical Sciences, Lucknow 226014, India; ^2^Department of Bone Marrow Transplantation and Cancer Immunotherapy, Hadassah-Hebrew University Medical Center, Ein Kerem, Jerusalem, Israel; ^3^Faculty of Medicine, Hebrew University of Jerusalem, Israel

## Abstract

Mesenchymal stem cell (MSC) therapy is an innovative approach in diabetes due to its capacity to modulate tissue microenvironment and regeneration of glucose-responsive insulin-producing cells. In this study, we investigated the role of MSC-derived exosomes in pancreatic regeneration and insulin secretion in mice with streptozotocin-induced diabetes. Mesenchymal stem cells (MSCs) were isolated and characterized from umbilical cord blood (UCB). Exosomes were isolated and characterized from these MSCs. Diabetes was induced in male C57Bl/6 mice by streptozotocin (STZ; 40 mg/kg body weight, i.p.) for five consecutive days. The diabetic mice were administered (i.v.) with MSC (1 × 10^5^ umbilical cord blood MSC cells/mice/day), their derived exosomes (the MSC-Exo group that received exosomes derived from 1 × 10^5^ MSC cells/mice/day), or the same volume of PBS. Before administration, the potency of MSCs and their exosomes was evaluated in *vitro* by T cell activation experiments. After day 7 of the treatments, blood samples and pancreatic tissues were collected. Histochemistry was performed to check cellular architecture and *β* cell regeneration. In body weight, blood glucose level, and insulin level, cell proliferation assay was done to confirm regeneration of cells after MSC and MSC-Exo treatments. Hyperglycemia was also attenuated in these mice with a concomitant increase in insulin production and an improved histological structure compared to mice in the PBS-treated group. We found increased expression of genes associated with tissue regeneration pathways, including *Reg2*, *Reg3*, and *Amy2b* in the pancreatic tissue of mice treated with MSC or MSC-Exo relative to PBS-treated mice. MicroRNA profiling of MSC-derived exosomes showed the presence of miRs that may facilitate pancreatic regeneration by regulating the Extl3-Reg-cyclinD1 pathway. These results demonstrate a potential therapeutic role of umbilical cord blood MSC-derived exosomes in attenuating insulin deficiency by activating pancreatic islets' regenerative abilities.

## 1. Introduction

Diabetes is a major growing health problem that has been estimated to afflict well over 300 million people globally. The two most common types of diabetes are type 1 (T1DM) and type 2 diabetes mellitus (T2DM). Together, they account for a large and growing patient population with pancreatic *β* cell deficiency. In the case of T1DM, *β* cell deficiency is associated with autoimmune destruction of *β* cells, while in the case of T2DM, it is associated with *β* cell dysfunction [1, 2]. Nevertheless, substantial *β* cell loss results in permanent endocrine deficiency and irreversible diabetes due to their limited regenerative capacity in adults [3]. Thus, any treatment modality to restore *β* cell mass would be beneficial for all forms of diabetes [4, 5]. Mesenchymal stem cells (MSCs) present a promising approach for restoring *β* cell mass in diabetes [6, 7]. Immune modulatory properties of MSCs have been attributed to their therapeutic effects in T1DM [8, 9]. Bone marrow-derived MSCs have been found to inhibit T cell proinflammatory response to islet antigen GAD65, in patients with T1DM [10, 11]. Moreover, these MSCs were shown to affect the antigen-presenting function of dendritic cells (DC) by promoting an immature DC phenotype [12]. The immunomodulatory effects of autologous bone marrow MSCs in patients with recent onset T1DM showed preservation of C peptide levels in response to a mixed meal tolerance test (MMTT) [13]. Similar immunomodulatory potential of MSCs derived from other sources, such as umbilical cord blood (UCB), will have a profound impact in developing therapeutic modalities for diabetes due to their availability and noninvasive nature of collection [14–16]. The mechanisms of action of MSCs have been attributed to their secretome rather than to their transdifferentiation [17]. However, MSC administration for therapeutic purposes may expose individuals to the side effects of cell-based therapy and immune rejection [18]. This has geared a great scientific interest in exploring alternative approaches inspired by the paracrine hypothesis in regenerative medicine, namely, using MSC-derived extracellular vesicles, such as exosomes, rather than the cells themselves. Exosomes have been shown to play a key role in cell-to-cell communication. Ample of data suggests that exosomes may transfer beneficial outputs from cells to neighboring diseased or injured cells through the delivery of biomolecules with therapeutic potential. The protective effect of UCB-derived MSC-secreted exosomes has been studied in myocardial repair and autoimmune diseases [14, 16]. However, the potential of UCB-MSCs and their derived exosomes in attenuating endocrine deficiencies caused due to nonautoimmune pancreatic *β* cell loss needs to be tested. In this study, we aimed to understand the therapeutic effect of exosomes secreted by UCB-derived MSC on pancreatic repair/regeneration in diabetic mice, where streptozotocin-induced pancreatic beta cell destruction leads to diabetes.

## 2. Materials and Methods

### 2.1. Isolation and Characterization of Human UCB-Derived MSCs

MSCs were isolated from human UCB as previously described [17]. The protocol has been approved by Institutional Committee for Stem Cell Research (IC-SCR; Code: 2019-03-IMP-08). MSCs propagated to the 3^rd^-8^th^ passages were characterized using accepted MSC-positive markers anti-CD73, CD90, and CD105 (BioLegend, San Diego, CA, USA), negative hematopoietic markers anti-CD45 and CD34 (BioLegend, San Diego, CA, USA), HLA-DR (BD Biosciences, San Jose, CA, USA) by confocal microscopy (FluoView F10i confocal microscope, Olympus), and flow cytometry (Becton Dickinson, New Jersey, USA). Negative gates were set relative to isotype controls. No chromosomal aberrations were observed at high passage (data not shown). *In vitro* differentiation was performed using StemPro™ Adipogenesis Differentiation Kit (A1007001, Thermo Scientific, Pittsburg, PA Scientific, USA), StemPro™ Osteogenesis Differentiation Kit (A1033201, Thermo Scientific, Pittsburg, PA Scientific, USA), and StemPro™ Chondrogenesis Differentiation Kit (A1007101, Thermo Scientific, Pittsburg, PA Scientific, USA) [19, 20].

### 2.2. Isolation and Characterization of Exosomes from Human UCB-Derived MSCs (MSC-Exo)

Exosomes were enriched by differential centrifugation as described previously [21]. Briefly, samples were centrifuged at 17,000*g* for 10 min at 4°C followed by ultracentrifugation (Beckman Coulter LE80K, CA, 362, USA) at 126,000*g* for 2 h at 4°C. The exosomal pellet was resuspended in an isolation buffer (PBS). Isolated exosomes were characterized for size using dynamic light scattering (DLS) analysis. The sample temperature was allowed to equilibrate for 10 min before each measurement and was shaken for 20 min at 37°C to dissolve any aggregation, followed by DLS measurements at 20°C using a Zetasizer Nano (Malvern Instruments Ltd., UK). The light scattering was recorded for 200 s with 10 replicate measurements. DLS signal intensity was calculated by the Dispersion Technology Software v.5.10 (Malvern Instruments Ltd., UK). The mean hydrodynamic diameter of exosomes was calculated by fitting a Gaussian function. The peak maximum of the Gaussian function was used to estimate exosome size. Gaussian fitting, mean value, and standard deviation were calculated and compared using Origin Pro 9.0.0 (Origin Lab Corp, USA). Shape characterization was done by scanning electron microscopy (SEM). MSC-Exo suspensions were mixed 1 : 1 with 4% paraformaldehyde and were then applied to 200-mesh nickel grids. They were dried and coated with gold particles, and images were taken by scanning electron microscope (JSM 6490).

T cell activation analysis was performed by using mouse spleen (source of T cell) and followed by CD3^+^ T cell enrichment column (cat MTCC-25, R&D systems, USA) as per the manufacturer's instruction. Proliferation analysis was performed on (i) unactivated T cells, (ii) activated T cells, (iii) activated T cells treated with MSCs, and (iv) activated T cells treated with MSC-Exo. For this, 1 × 10^5^ T cells were seeded in 10% FBS (exosome free) RPMI media in 96-well plates. Cells were activated by using 50 *μ*l of 5 *μ*g/ml anti-CD3 antibodies (or 2C11) (BioLegend, San Diego, CA, USA) for 4 days at 37°C. Similarly, in the treated group, these activated T cells were cocultured with MSCs (5 × 10^4^ cells/well) and MSC-derived exosome (collected from 5 × 10^4^ MSCs cells for 24 h). After 4 days, nonadherent T cells were collected and live T cell counting was performed by using trypan blue and haemocytometer.

### 2.3. Microarray Analysis for MicroRNA Profile

For microarray analysis, RNA was isolated from MSC and MSC-Exo by miRNAeasy kit (Qiagen India Pvt. Ltd.) as per the manufacturer's protocol. The quantity was checked by NanoDrop ND-2000 (Thermo Fisher Scientific, Pittsburgh, PA). Samples with A260/280 more than 1.8 were subjected to microarray analysis by GeneChip™ miRNA 4.0 Array according to the manufacturer's instruction (Thermo Fisher Scientific). Transcriptome Analysis Console program (version 4.0.0.25, Applied Biosystems) conducted a statistical analysis of the output files (.CEL). Conditions used were background correction, quantile, normalization, description, and log_2_ value conversion using the RMA+DABG algorithm. Data represented as heat map and volcano plot.

### 2.4. *In Vivo* Diabetic Mouse Model Development

**Figure 1:**
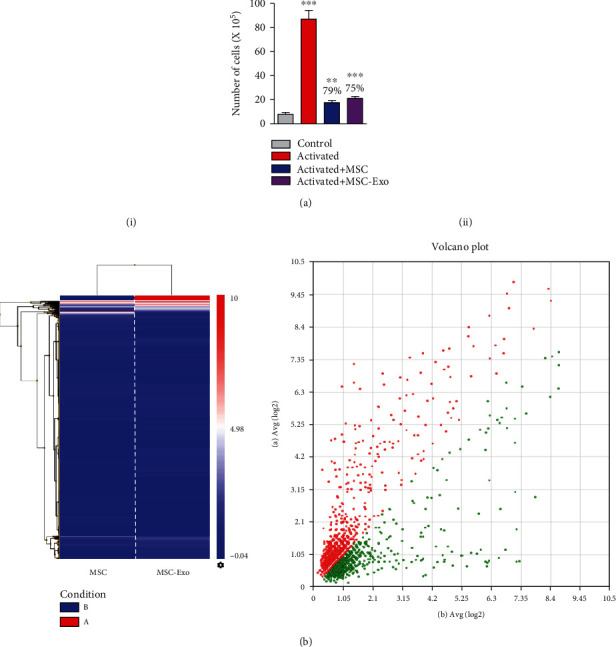
Human UCB-MSC and their derived exosomes showed similar microRNA profile and function. (a) Histogram representing T cell activation or proliferation *in vitro* attenuated after treatment with MSCs and MSC-Exo. Data are shown as the mean ± SD. The significance of differences between mean values was estimated by Student's *t*-test (unpaired, two-tailed). ^∗^*p* < 0.05 and ^∗∗∗^*p* < 0.001 compared to control. (b) (i) Heat map and (ii) volcano plot representing the comparative miRNA profile of MSCs and MSC-Exo.

8-week-old male C57Bl/6 mice (body weight~20 g) were used throughout the study. The protocol has been approved by Institutional Animal Ethics Committee (Ref. no: P-03/20/2017). Mice were procured from CSIR-Indian Institute of Toxicology Research, Lucknow, and were housed at the animal care facility of Sanjay Gandhi Postgraduate Institute of Medical Science, Lucknow. All mice were housed under the specific pathogen-free conditions in individually ventilated cages with 12 h light and dark cycle. All mice received drinking water and diet *ad libitum*. To induce T1DM, mice were injected intraperitoneal with multiple-dose injections of streptozotocin (STZ) 40 mg/kg body weight, freshly dissolved in 0.1 mmol/l sodium citrate (pH 4.5) for 5 consecutive days as previously described [22]. Blood samples for glucose levels monitoring were collected daily (from day 1 to 10) from tail vein using a glucometer (Elite Diabetes Care System). Animals were considered diabetic when their blood glucose levels were ≥200 mg/dl. The control nondiabetic mouse group received a corresponding volume of 0.1 mmol/l of sodium citrate (pH 4.5) (Figure 1). Insulin was measured on pancreatic tissue using mouse insulin ELISA kit (Thermo Scientific, Pittsburg, PA Scientific, USA) on day 10 and day 17 in the control and diabetic mice treated with PBS, MSCs, or MSC-Exo (*n* = 6/group).

### 2.5. Proliferation Assays in Diabetic Mice Administered with MSCs and MSC-Exo

After 10 days of STZ treatment, mice were randomly divided into three groups: (i) vehicle group that received i.v. PBS injections from day 11 to day 17 (*n* = 6), (ii) MSC group that received i.v. injection of 1 × 10^5^ MSC cells/mice from day 11 to day 17 (*n* = 6), (iii) Exo group that received i.v. injection of MSC-Exo derived from 1 × 10^5^ MSCs/mice from day 11 to day 17 (*n* = 6). To investigate the homeostatic turnover of pancreatic cells in all three groups, two doses of BrdU (at 2 h and 16 h) were administered at day 18 (three mice for each group, BrdU at 50 *μ*g/kg in saline, i.p.) (Sigma-Aldrich, St. Louis, Missouri, USA).

### 2.6. Histology

Mice were euthanized on day 18 by cervical dislocation. For excision of the pancreas, mice were perfused with 1x PBS. Pancreases were carefully cleaned to remove excess fat tissue, fixed in 4% buffered paraformaldehyde (PFA) and then embedded in paraffin wax for histological processing. Paraffin-embedded tissues were sectioned (5 *μ*m thickness) using a microtome (Thermo Scientific, Pittsburg, PA Scientific, USA). The sections were then processed for histological and/or immunohistochemical analysis.

For histopathogical analysis, five random fields were selected on three different sections from each group and the number of cells were counted as healthy and damaged on the basis of cellular morphology.

### 2.7. Proliferation Assay

Proliferating cells in pancreas sections were detected by indirect immunoperoxidase test as described previously [23]. Briefly, the sections (5 *μ*m thickness) were deparaffinized using xylene and rehydrated by descending gradings of ethanol. The sections were washed in 1x PBS and were further processed for heat-mediated antigen retrieval by using antigen-unmasking solution (Vector Labs, USA). The sections were washed with 1x PBS followed by blocking with 3% bovine serum albumin (BSA, Sigma-Aldrich, St. Louis, Missouri, USA). The sections were then probed with anti-BrdU antibody (B2531, Sigma-Aldrich, St. Louis, Missouri, USA) overnight at 4°C. In the next morning, the sections were washed with 1x PBS and incubated with HRP-tagged anti-mouse secondary antibody for 1 h at 37°C. The sections were then washed for three times with 1x PBS, and color was developed by 3,3′-diaminobenzidine (DAB) chromogen solution (106038, GeNei™) at 37°C for 10 min. Sections were counterstained with haematoxylin, dehydrated in ethanol gradings, and mounted in DPX. Images were captured on an Olympus IX73 microscope and analysed by ImageJ software.

### 2.8. Western Blotting

Exosomes were lysed in RIPA buffer with a freshly added protease inhibitor cocktail (Roche Diagnostics, Germany). Protein concentrations were determined by BCA (Pierce, BCA Protein Assay Kit, Thermo Scientific, Pittsburg, PA). Equal amount of proteins was denatured and resolved on denaturing SDS-PAGE gel (12%) and transferred to PVDF membrane. To prevent any protein loss due to stripping and for the ease of interpretation, membrane was cut into the lane after transfer of gel. The membrane was probed overnight with mouse anti-TSG101 (ab125011, Abcam, Cambridge, MA, USA), anti-CD63 (ab59479, Abcam), and anti-CD81 (ab109201, Abcam, Cambridge, MA, USA) antibody followed by HRP-conjugated secondary antibody. The membrane was visualized by an enhanced chemiluminescence detection system (Clarity™ western ECL substrate). Images were acquired on a ChemiDoc imaging system (Universal Hood III, Bio-Rad, California, USA).

### 2.9. Next-Generation Sequencing for Transcriptome Profile

RNA was extracted from diabetic mouse pancreas treated with PBS, MSCs, and MSC-Exo for sequencing and qPCR experiments. The RNA quality was tested using 1% agarose gel electrophoresis, quantities were measured by Qubit system (Invitrogen), and absorbance ratios (A260/280 and A260/230) were tested using NanoDrop ND-2000 (Thermo Fisher Scientific, Pittsburgh, PA, USA). Samples with A260/280 and A260/230 ratios more than 2.0 were used for sequencing using a MinION sequencer according to the manufacturer's instructions (Oxford Nanopore Technologies). In brief, the RNA samples (50 ng per sample) were reverse transcribed with a cDNA-PCR sequencing kit (SQK-PCS109); then, the samples were barcoded with a PCR barcoding kit (SQK-PBK004). Samples were primed with a flow cell priming kit (FLP001) and loaded on MinION flow cells, according to the manufacturer's instruction (Oxford Nanopore Technologies). MinKNOW was used to convert the generated Fast_5 files into FastQ format. Fold expressions were summarised in a heat map using HeatMapper software (http://www.heatmapper.ca/). Pathway analysis was performed using Reactome (https://reactome.org/), KEGG (http://www.genome.jp/kegg/), and Panther (http://pantherdb.org/).

### 2.10. Real-Time PCR

cDNA synthesis was performed with 1 *μ*g of total RNA template per sample (the PBS, MSC, and MSC-Exo groups) by cDNA RT kit (Thermo Scientific, Pittsburgh, PA, USA). The protocol was used according to the manufacturer's instructions. Real-time PCR was performed using an ABI 7500 Sequence Detection System (Applied Biosystems, California, USA) in the presence of SYBR Green Master Mix (Takara Bio, Shiga, Japan). Standard PCR conditions were used as prescribed in the reagent protocol.

The primer sequences used were as follows: *Reg3* forward primer-5′-GAATATACCCTCCGCACGCA-3′ and *Reg3* reverse primer-5′-TCTTTTGGCAGGCCAGTTCT-3′, *Reg2* forward primer-5′-AATCAACTGCCCAGAGGGTG-3′ and *Reg2* reverse primer-5′-GCCACAAAGTTGCTCTCAGC-3′, *Amy2b* forward primer-5′-TGGGAGGACTGCTATTGTCC-3′ and *Amy2b* reverse primer-5′-CATTGTTGCACCTTGTCACC-3′, *TLR4* forward primer-5′-ATGCATGGATCAGAAACTCAGCAA-3′ and *TLR4* reverse primer-5′-AAACTTCCTGGGGAAAAACTCTGG-3′, and 18S forward primer-5′-GGCCCTGTAATTGGAATGAGTC-3′ and 18S reverse primer-5′-CCAAGATCCAACTACGAGCTT-3′. Gene expression was calculated relative to the endogenous control sample (18S). Fold change was calculated relative to the PBS control group using the 2^−ΔΔCt^ method (where Ct is the threshold cycle).

### 2.11. Statistical Analysis

GraphPad Prism 5 software was used for the statistical analysis. Data are presented as the mean ± standard deviation. The significance of differences between mean values was estimated using two-tailed unpaired Student's *t*-test. The statistical significance was set at ^∗^*p* < 0.05, ^∗∗^*p* < 0.01, and ^∗∗∗^*p* < 0.001 vs. their respective control.

## 3. Results

### 3.1. Immunophenotypic Characterization and Differentiation of Human UCB-Derived MSCs

**Figure 2:**
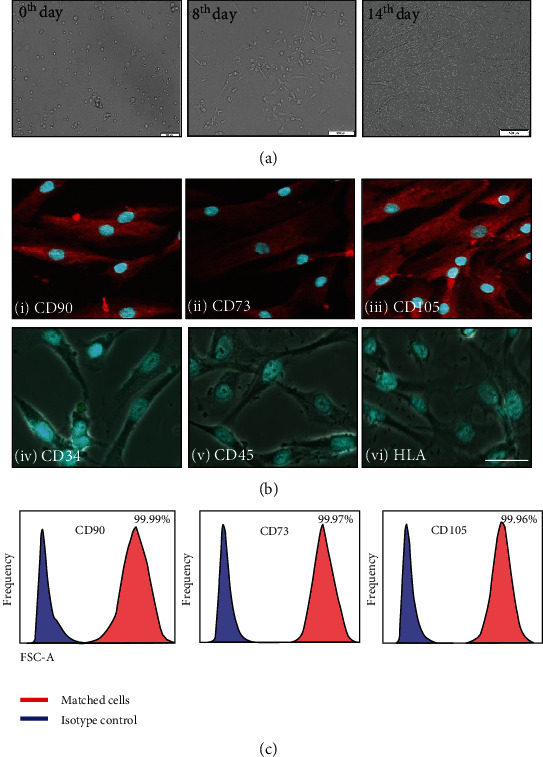
Characterization of human UCB-derived mesenchymal stem cells (MSCs). (a) Typical fibroblastic morphology of MSCs (i) at day of seeding, (ii) at day 8, and (iii) at day 14 of seeding. Scale bar = 500 *μ*M. (b) Confocal microscopy images showing expression of (i) CD90, (ii) CD73, and (iii) CD105 in isolated MSCs and lack of expression of (iv) CD34, (v) CD45, and (vi) HLA-DR in isolated MSCs. Scale bar = 100 *μ*M. (c) Flow cytometry showed expression of typical MSC surface markers CD90, CD73, and CD105. FSC-A: forward scattering area.

**Figure 3:**
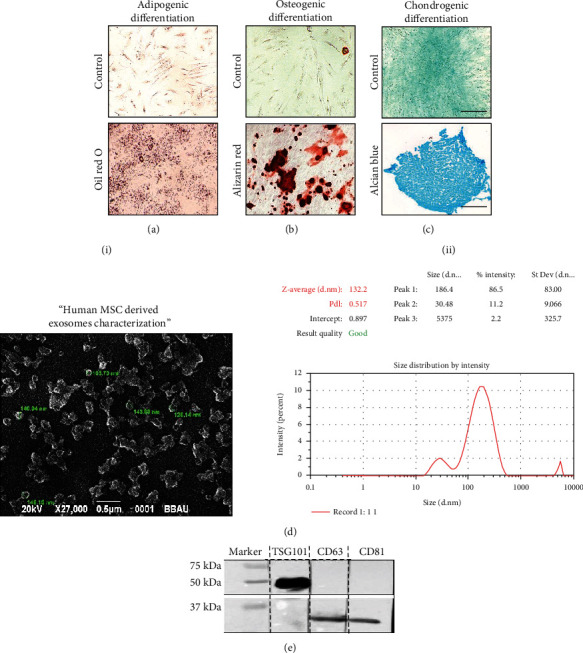
Characterization of MSCs and MSC-Exo. Representative photomicrographs of human umbilical cord blood-derived mesenchymal stem cells differentiated into (a) adipocytes, (b) osteocytes, and (c) chondrocytes and stained with Oil Red, Alizarin Red, and Alcian Blue stain, respectively. Scale bar = 100 *μ*M. (d) (i) Electron micrograph of an MSC-Exo showing that 92.7% of the exosomes were between 50 and 140 nm (indicated by green letter). Scale bar = 0.5 *μ*M. (ii) MSC-derived exosome size ranging between 100 and 140 nM by dynamic light scattering analysis. (e) Representative immunoblot showing exosome-specific markers TSG101, CD63, and CD81 in isolated exosomes. Three independent experiments were performed. Full blot is submitted as a supplementary file.

MSCs isolated from human UCB (hUCB) were identified by their fibroblast spindle-shaped morphology (Figure 2(a)). Expressions of typical MSC markers (Figure 2(b, i–iii)) and absence of staining for hematopoietic markers (Figure 2(b, iv–vi)) were observed using immunofluorescence. The results were confirmed by FACS analysis for the presence of MSC markers CD73 (96%), CD90 (99%), and CD105 (99.9%) (Figure 2(c)). Differentiation assays performed on isolated MSCs exhibited as expected trilineage differentiation potential (Figures 3(a)–3(c)). Adipogenic differentiation was confirmed by the positive Oil Red O staining of neutral lipid vacuoles in the cells on day 14 of culture. Under osteogenic differentiation media, the cells exhibited nodular calcium deposition, which was stained with alizarin red staining on day 21 of culture. Four weeks under chondrogenic conditions, the cells exhibited typical chondrocyte-like lacunae. Alcian Blue staining confirmed the presence of secreted glycosaminoglycans.

### 3.2. Human UCB-MSCs (MSC) and Their Derived Exosomes (MSC-Exo) Showed Similar MicroRNA Profile and Function

Isolated exosomes were characterized by their cup-shaped spheroidal morphology (size: ~132 nm) (Figure 3(d, i and ii)) and the presence of exosome-specific markers, i.e., TSG101, CD63, and CD81 proteins (Figure 3(e)). The heat map and volcano plots generated after microarray analysis showed a similar microRNA profile of MSC-Exo and MSCs (Figure 1(b, i and ii)). The expression patterns of miR-let 7a-5p, miR-24-3p, miR-2115-5p, miR-4156, miR-663a, miR-424-5p, miR-30d-5p, miR-450-p, miR-23b-5p, and miR19b-1-3p were similar between MSC-EXO and MSCs.

Suppressive effects of MSCs and their derived exosomes were tested on 2C11 antibody-activated T cells cocultured in the presence/absence of MSCs and MSC-Exo. 2C11 antibody was shown to induce T cell proliferation which was significantly attenuated by MSC-Exo (*p* < 0.05) and MSCs (*p* < 0.05) treatments, compared to control (Figure 1(a)).

### 3.3. *In Vivo* Mouse Model Induction of Diabetes and Pancreatic Injury by STZ Treatment

**Figure 4:**
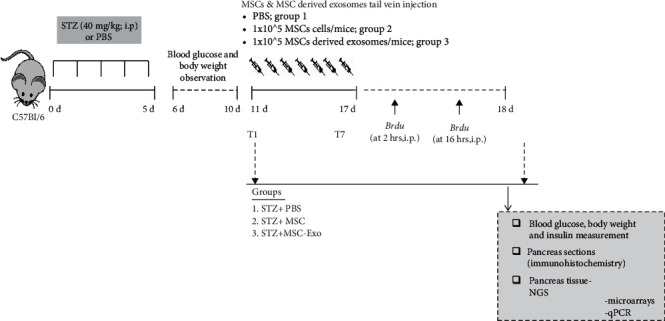
Timeline of the *in vivo* experimental design. Schematic illustration of the experimental plan. (a) In brief, 8-week-old adult male C57BL/6 mice (body weight~20 g) were used throughout the study. To induce diabetes, mice were injected with multiple low-dose injections of streptozotocin intraperitoneal (i.p.) (STZ; 40 mg/kg freshly dissolved in 0.1 mmol/l sodium citrate (pH 4.5)) for 5 consecutive days. Blood samples were collected from tail-vein and were used to monitor glucose levels. Animals were considered diabetic when their blood glucose levels were ≥200 mg/dl. Mice in the nondiabetic control group received a corresponding volume of sodium citrate buffer alone. On day 10, after STZ treatment, mice were randomly divided into three groups: (i) STZ+PBS group that received i.v. injection of PBS (*n* = 6), (ii) STZ+MSC group that received i.v. injection of 1 × 10^5^ UCB-MSC cells/mice (*n* = 6), and (iii) STZ+MSC-Exo group that received i.v. injection of exosomes derived from 1 × 10^5^ MSCs/mice from day 11 to day 17 (*n* = 6). To explore the homeostatic turnover of pancreatic cells, two doses of 50 *μ*g/kg BrdU (at 2 h and 16 h) were administered at day 18 (three mice for each group). At day 18, mice were sacrificed and processed biochemical and molecular analysis.

**Figure 5:**
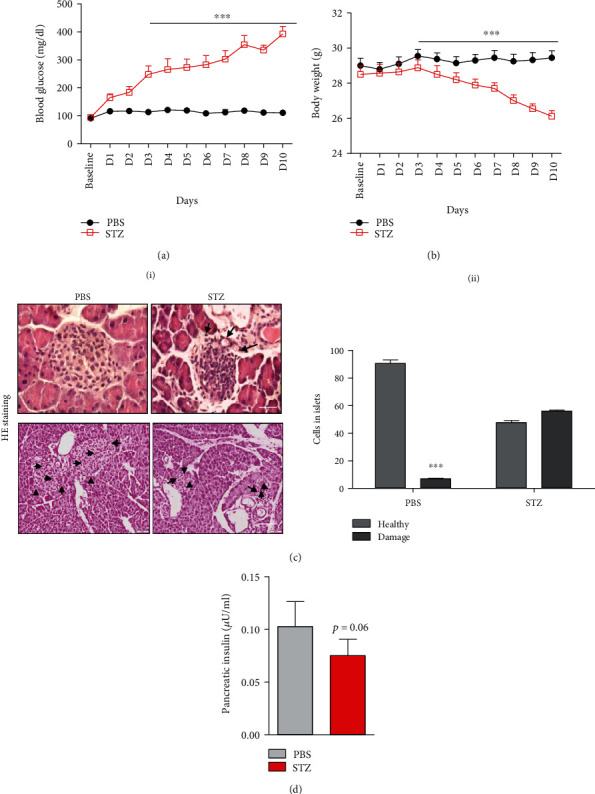
STZ treatment-induced diabetes in mice. (a) Line graph showing blood glucose levels in diabetic mice compared to the control animals over the experiment period (*n* = 6/group). (b) Line graph showing body weight in the diabetic group compared to the control group (*n* = 6/group). (c) (i) Representative images of histological analysis of H&E (scale bar = 20 *μ*M) in the diabetic and control groups showing the altered pancreatic morphology. The lower panel shows images of H&E staining with multiple islets (low magnification, scale bar = 100 *μ*M). (c) (ii) Histogram representing the number of healthy and damaged cells in islets counted on three different sections (five random fields) in the diabetic group and control group. (d) Histogram representing insulin levels in pancreatic tissue of the diabetic and control groups. Data are shown as the mean ± SD (*n* = 6/group). The significance of differences between mean values was estimated by nonparametric Student's *t*-test (unpaired, two-tailed). ^∗^*p* < 0.05 and ^∗∗∗^*p* < 0.001 compared to control animals.

The schematic study design is shown in Figure 4. STZ-injected animals had significantly higher blood glucose as compared to vehicle-injected animals' hours of the initial STZ injection (*p* < 0.001; Figure 5(a)). Body weight decreased in STZ-treated mice from day 5^th^ of STZ treatment; the decrease was significant at days 9 and 10 relative to their baseline (Figure 5(b)). Histopathological analysis showed loss of distinct lobular and cellular boundaries, vacuolar degeneration, fibrosis, and marked decrease in islets of Langerhans size as well as decrease number of cells in islets of Langerhans in the diabetic group compared to the nondiabetic group (Figure 5(c, i)). The number of damaged cells was increased in the diabetic group compared to the control (Figure 5(c, ii)). Pancreatic insulin content as estimated by ELISA showed significantly lower levels in diabetic animals, relative to nondiabetic animals (Figure 5(d)).

### 3.4. MSC-Exo Attenuated Hyperglycemia and Pancreatic Injury in STZ-Induced Diabetic Animals

**Figure 6:**
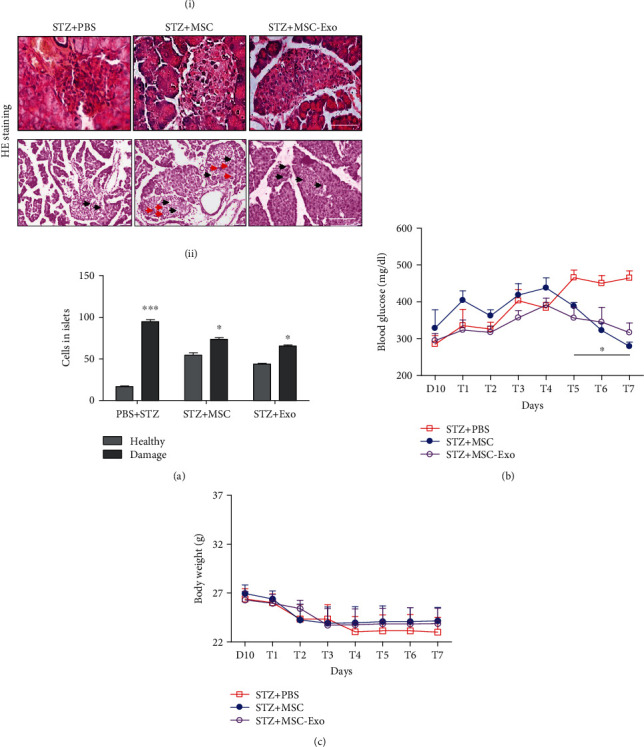
Reduced glucose levels in the MSC and MSC-Exo-treated groups. (a) (i) Representative images of H&E staining (scale bar = 50 *μ*M) of pancreatic tissue of the STZ+MSC and STZ+MSC-Exo groups compared to the STZ+PBS group. The lower panel shows images of H&E staining with multiple islets (low magnification, scale bar = 100 *μ*M). (ii) Histogram representing number of healthy and damaged cells in islets counted on three different sections (five random fields) in the STZ+PBS, STZ+MSC, and STZ+MSC-Exo groups. (b) Line graph showing blood glucose level in diabetic animals treated with the MSC and MSC-Exo groups compared to the PBS-treated group. (c) Line graph showing body weight in diabetic animals treated with the MSC and MSC-Exo groups compared to the PBS-treated group. Data are shown as the mean ± SD (*n* = 6/group). The significance of differences between mean values was estimated by nonparametric Student's *t*-test (unpaired, two-tailed) ^∗^*p* < 0.05 and ^∗∗∗^*p* < 0.001 compared to the PBS-treated group.

After 10 days of STZ injection, diabetic animals were randomly assigned into three treatment groups: PBS, MSCs, and MSC-Exo (Figure 4). The treatment was given for 7 consecutive days (T1 to T7). A reduction in blood glucose levels was observed after MSCs or MSC-Exo treatment from day 1 of treatment (T1) onwards; the reduction was found to be significant from day 5 of treatment (T5) compared to their baseline (Figure 6(b)). In PBS-treated diabetic mice (STZ+PBS group), blood glucose further rose from day 1 of treatment (T1) and remained significantly higher compared to MSC/MSC-Exo-treated animals till day 7 (T7) (Figure 6(b)). Body weights did not change significantly during the treatment period (from T1 to T7, Figure 6(c)). Histological analysis showed recovery of the cellular architecture of islets of Langerhans, with the reduction in vacuoles and fibrosis in the MSC-Exo and MSC-treated group as indicated by the increased number of cells (Figure 6(a, ii)) and cellular density in islets of Langerhans compared to the PBS-treated group (Figure 6(a, i)). Although the boundaries of islets of Langerhans were not well defined, they were surrounded by a fine capsule with increased cellular density in diabetic mice treated with MSCs and MSC-Exo (Figure 6(a)).

### 3.5. MSC-Exo-Induced Proliferation of Islet Cells and Increased Pancreatic Insulin Production in Diabetic Animals

**Figure 7:**
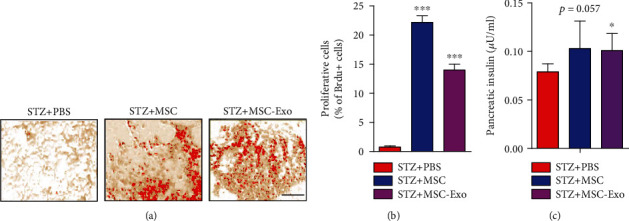
Intravenous post-MSC and Exo treatments increase cell proliferation and insulin level in the MSC and MSC-Exo-treated group. (a) Representative histological images of pancreatic tissue stained with BrdU showing increased BrdU-positive cells in diabetic animals treated with MSC or MSC-Exo in comparison with PBS-treated animals. Scale bar = 100 *μ*M. (b) Histogram representing the percentage of BrdU-positive cells in MSC and MSC-Exo-treated diabetic animals in comparison with the PBS-treated group. (c) Histogram representing increased insulin level in pancreatic tissue of diabetic animals treated with MSC and MSC-Exo in comparison with PBS-treated animals. Data are shown as the mean ± SD (*n* = 6/group). The significance of differences between mean values was estimated by nonparametric Student's *t*-test (unpaired, two-tailed). ^∗^*p* < 0.05 and ^∗∗∗^*p* < 0.001 compared to the PBS-treated group.

Proliferation induction in the pancreatic islets was observed in MSC and MSC-Exo-treated animals compared to the PBS-treated group (Figure 7(a)), as indicated by increased BrdU-positive cells in islets of Langerhans (*p* < 0.001 vs. PBS group; Figure 7(b)). Moreover, the pancreatic insulin content (ELISA assay) estimated at the end of treatment (at T7) was significantly higher in the MSC and MSC-Exo-treated groups relative to the PBS group (*p* = 0.057 vs. PBS group; Figure 7(c)).

### 3.6. Regeneration Pathways Were Differentially Regulated in the Pancreas of MSCs and MSC-Exo-Treated Animals

**Figure 8:**
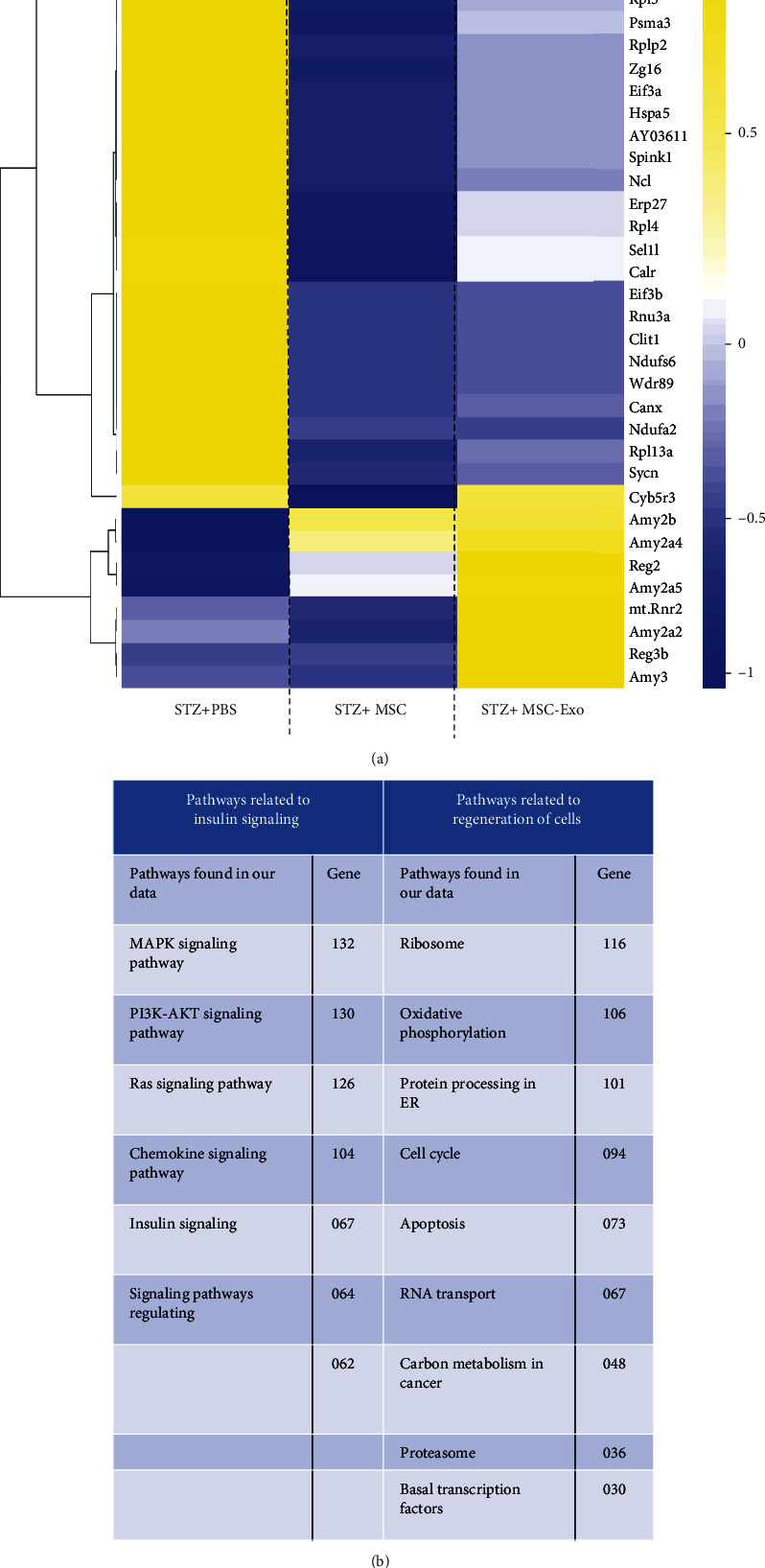
Differential gene expression in pancreatic tissue of animals treated with MSCs and MSC-Exo. (a) Heat map representing differential gene expression in the pancreatic tissue of diabetic animals treated with MSCs and MSC-Exo in comparison with PBS-treated animals. *p* < 0.05 was considered as the level of significance, and ±2-fold change was the cut off value for heat map generation. (b) The table represents significantly differentially regulated pathways.

NGS analysis revealed out 43,000 genes, 667 differentially up and downregulated genes (cut off > ±2.0 fold change), in the pancreatic tissue of diabetic animals treated with MSCs and MSC-Exo relative to the PBS group. The expressions of these differentially regulated genes were summarised in a heat map (Figure 8(a)), and pathway analysis of the differentially regulated genes showed involvement of insulin signaling and tissue regeneration pathways.  Figure 8(b) shows genes, identified in our analysis, that have been associated with these pathways. A few of the identified genes were further validated using qPCR analysis, including *Reg3* (regenerating islet-derived protein III), *Reg2* (regenerating islet-derived protein II), and *Amy2b* (amylase Alpha 2b) and *TLR4* (Supplementary Table 1).

## 4. Discussion

Restoring *β* cell mass is an important management modality in diabetes [3–5]. Among several strategies for restoring *β* cell mass, MSCs might serve as a promising agent due to their unique properties, including conditional differentiation, tropical support, and capabilities of spontaneous differentiation into connective tissue [24]. Moreover, they possess intrinsic immunosuppressive abilities [25]. MSCs derived from several sources such as bone marrow and dental pulp have been reported to attenuate diabetes and have pancreatic islet regeneration ability [26, 27]. Currently, the therapeutic potential of MSCs was extracted from studies based on the animal models; however, four clinical trials of MSCs in T1DM are known till date, but due to smaller sample size, these results cannot be adequately evaluated and they are unable to demonstrate the significance as well [28]. Along with these, therapeutic use of MSCs may lead to cell-based therapy side effects and immune rejection. Recent studies [29–31] reported the promising role of exosomes in the treatment of T1DM. These studies suggested that MSC-derived exosome could be a better alternative to MSCs due to their fewer side effects [29–31]. However, future studies and clinical trials are required to understand their mechanism of action. Moreover, the potential of MSC and MSC-derived exosomes in attenuating endocrine deficiencies caused by nonautoimmune pancreatic *β* cell loss needs to be tested. With this aim, we isolated and characterized MSCs from h-UCB (a noninvasive source of stem cells) based on their morphology, surface markers, and differentiation ability. After successful isolation of MSCs, we isolated the MSC-derived exosomes. Functional characterization of the MSC and MSC-derived exosomes was confirmed in vitro, where activated T cells were suppressed after treatment with MSC (79%) or MSC-Exo (75%). A similar report also showed the effect of MSC-Exo over MSCs in the rat model of T1DM [31]. Moreover, a therapeutic potential of stem cell-derived exosomal miRNA has been reported. Since the miRNA profiles of exosomes vary with type of stem cells, we compared the regulatory profile of these two treatment strategies (MSC and MSC-Exo) by comparing their miR profile. Using microarray analysis, we observed that hUCB-derived MSCs and their exosomes share similar miRNA profiles. We noted a huge expression of miRs that were known to be associated with cellular proliferation, cell cycle, inflammation, apoptosis, and metabolism pathways.

After successful isolation and characterization of MSC and MSC-Exo, we assessed their effect on pancreatic regeneration and the associated mechanism of action *in vivo*. STZ-induced diabetes and pancreatic injury were confirmed, respectively, by the induction of hyperglycemia and loss of pancreatic cellular architecture (diminution of islets of Langerhans' size and insulin content) compared to the control mice. Similar to our observation, Sabry et al. [31] reported high inflammation, vacuolar degeneration, congestion, and fibrosis along with the marked decrease in the size of islets of Langerhans. In our study, we have demonstrated regenerative benefits of MSCs derived from human UCB, in STZ-induced diabetic mice. We found that both MSCs and MSC-Exo had similarly attenuated diabetes, as evidenced by decreased blood glucose levels after MSCs and MSC-Exo treatments. The treatments also promoted pancreatic regeneration compared to vehicle-treated diabetic mice. In support of our observations, study in rat pancreas demonstrated marked recovery of pancreatic architecture after treatment with MSC and MSC-Exo [31]. A similar beneficial effect of MSC-Exo, on STZ-induced diabetes, has also been reported in a recent study [32]. The author suggests that exosomes promote insulin sensitivity, increase glucose uptake and metabolism in peripheral tissues, and protect pancreatic islets from damage by inhibiting STZ-induced *β* cell apoptosis. Recently, Mahdipour et al. have shown that exosomes derived from menstrual blood MSCs can regulate *β* cell regeneration through PDX1-dependent mechanism in T1DM rat model [33].

Though the studies have confirmed the beneficial effect of MSC and MSC-Exo on pancreatic regeneration, but the mechanism remained illusive. Here, we performed transcriptome profiling in diabetic, MSC-, and MSC-Exo-treated pancreas. During transcriptome analysis of the pancreatic tissues, most of the transcripts (~80%) were found to be similarly regulated in MSCs and the MSC-Exo-treated group, relative to the PBS-treated group. However, a few of the transcripts were differentially regulated between MSCs and the MSC-Exo-treated group. For example, *Cyb5r3* (NADH cytochrome b5 reductase (b5R)), *Reg2* (regenerating islet-derived protein II), *Reg3b* (regenerating islet-derived protein 3b), *mtRnr2* (mitochondrial encoded 16S rRNA2), Amy*2a5* (amylase alpha 2A5), *Amy2a2* (amylase alpha 2A2), and *Amy2a3* (amylase alpha 2A3) genes were upregulated in the MSC-Exo group, but not in the MSC-treated group, as compared to the PBS-treated group. Moreover, *Cela2a* (chymotrypsin-like elastase 2A), Amy*2a4* (amylase alpha 2A4), and *Amy2b* (amylase alpha 2b) genes were significantly upregulated in MSC-Exo relative to the MSC-treated group. The genes that regulated in the treated groups compared to the PBS-treated group majorly belong to insulin signaling and tissue regeneration pathways. Within the insulin signaling pathway, the highest number of genes was associated with the MAPK signaling (132 genes) and the PI3K-AKT pathway (130 genes), including Ras, MAPKs, AKT, and IRS, while tissue regeneration pathway includes genes associated with the following processes: oxidative phosphorylation (106 genes), ER protein processing (101 genes), cell cycle (94 genes), ribosomal pathway (116 genes, predominantly Rpl 2-13, Rps 1-30, and Mrps 1-12), and proteasomal pathway (36 genes, Psmb, Psmd, and Psme (proteosomal subunit)).

Furthermore, by using qPCR analysis, we validated the upregulation of *Reg3* (regenerating islet-derived protein III), *Reg2* (regenerating islet-derived protein 2), and *Amy2b* (amylase alpha 2B) in the pancreatic tissue of the MSC and MSC-Exo-treated groups, in comparison to the PBS-treated group. *Reg* gene encodes an endogenous lectin which may be involved in the regeneration and growth of *β* cells [34]. *Reg3* could also stimulate *β* cell regeneration by reducing inflammatory conditions by the Jak-Stat3 signaling pathway [34]. Thus, upregulation of *Reg3* found in the pancreas of MSC-Exo-treated diabetic animals may have a role in the *β* cell regeneration leading to the recovery of the insulin level [35–37]. Furthermore, an indispensable role of Reg protein in the pathophysiology of various human inflammatory diseases, especially *β* cell regeneration in pancreatic inflammation damage models, has been demonstrated [37]. Reg is regulated by multiple genes, including Glp1 [38], PDX1 [33], Extl3 [39, 40], and PARP [41] and in turn affects multiple signaling pathways in a stimulus-dependent manner. Reg3a has been shown to regulate keratinocyte proliferation and differentiation after skin injury via Reg-Extl3-PI3K-Cyclin D1 pathway [42], while Reg3g downregulates the Stat3-Socs signaling which may result in enhanced apoptosis in acute pancreatitis [43]. The pathways analysis in our study revealed that most of the downstream targets of *Reg2* and *Reg3* were altered in the pancreas of MSC-Exo-treated animals. Moreover, several miRNAs regulating these signaling pathways were found in the UCB-MSC-derived exosomes, including miRNA-regulating Extl3 (miR-let-7a-5p, miR-24-3p, and miR-19-b-1-5p) and Cela2a (miR-450-b-5p) expression. Thus, miRNAs in the MSC-derived exosomes may facilitate regeneration by regulating the Extl3-Reg-cyclinD1 pathway in the pancreas.

## 5. Conclusion

In conclusion, we have shown that exosomes derived from human UCB-MSCs significantly reduce blood glucose level, increase insulin production, limit pancreatic tissue damage, and improve disease outcomes in STZ-induced diabetic mice. Furthermore, exosome treatment-induced pancreatic tissue regeneration likely increased the Reg3 signaling pathways. Thus, UCB-MSC-derived exosomes may activate intrinsic regenerative abilities of pancreatic islets and attenuate insulin deficiency caused due to pancreatic cell destruction.

## Data Availability

The datasets generated during and/or analysed during the current study are available from the corresponding author upon reasonable request.

## Supplementary Materials

Supplementary materials Supplementary Table 1: qPCR analysis for *Reg2*, *Reg3*, *Amy2b*, and *TLR4*. MSC-Exo treatment increases *Reg2*, *Reg3*, and *Amy2b* expressions. Along with this, MSC and MSC-Exo treatment attenuated STZ-induced increase in the level of *TLR4* mRNA. Data represent as the mean ± SD; ^∗^*p* < 0.05 versus the STZ+PBS group, by a two-tailed unpaired *t*-test.
