# Supplementary material for: Exosomes Secreted by Umbilical Cord Blood-Derived Mesenchymal Stem Cell Attenuate Diabetes in Mice
Source: J Diabetes Res. 2021 Dec 10;2021:9534574. doi: 10.1155/2021/9534574 (PMC8683199; doi:10.1155/2021/9534574)
Supplement: Supplementary materials — Supplementary Table 1: qPCR analysis for Reg2, Reg3, Amy2b, and TLR4. MSC-Exo treatment increases Reg2, Reg3, and Amy2b expressions. Along with this, MSC and MSC-Exo treatment attenuated STZ-induced increase in the level of TLR4 mRNA. Data represent as the mean ± SD; ∗p < 0.05 versus the STZ+PBS group, by a two-tailed unpaired t-test. [file 9534574.f1.zip › Supplementary Figure.docx]

**Exosomes secreted by Umbilical cord blood-derived mesenchymal stem cell attenuates diabetes in mice.**

**Supplementary Figure**

Rajni Sharma^1*^, Manju Kumari^1*^, Suman Mishra^1^, Dharmendra K. Chaudhary^1^, Alok Kumar^1^, Batia Avni ^2,3^ and Swasti Tiwari^1#^

^1^ Department of Molecular Medicine & Biotechnology, Sanjay Gandhi Post Graduate Institute of Medical Sciences, Lucknow- 226014, India

^2^ Department of Bone Marrow Transplantation and Cancer Immunotherapy, Hadassah-Hebrew University Medical Center, Ein Kerem, Jerusalem, Israel.

^3^ Faculty of medicine, Hebrew University of Jerusalem, Israel

* Equal authorship

**Table: 1**


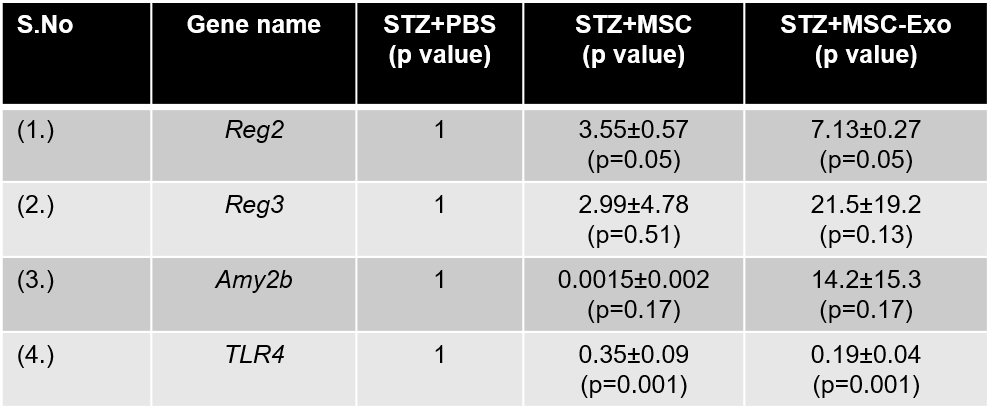


qPCR analysis for *Reg2, Reg3, Amy2b* and *TLR4*. MSC-Exo treatment increases

*Reg2, Reg3 and Amy2b expression. Along with this, MSC and MSC-Exo treatment attenuated STZ-induced increase in the level of TLR4 mRNA. Data represent as mean ± SD; *p < 0.05 versus STZ+PBS group, by tw*o tailed unpaired t-test.
