# Supplementary figures and images for: Exosomes Secreted by Umbilical Cord Blood-Derived Mesenchymal Stem Cell Attenuate Diabetes in Mice
Source: J Diabetes Res. 2021 Dec 10;2021:9534574. doi: 10.1155/2021/9534574 (PMC8683199; doi:10.1155/2021/9534574)

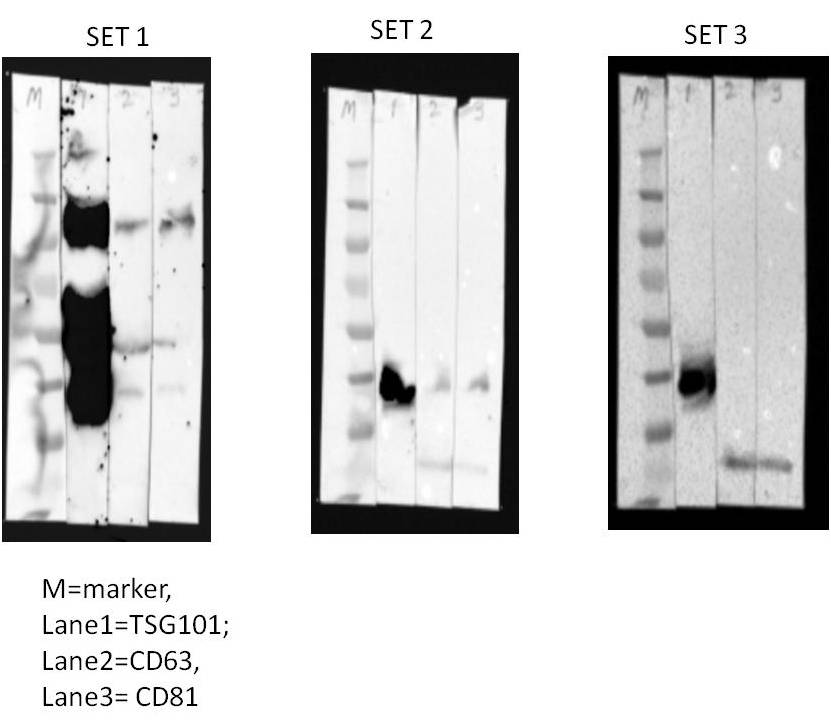

Supplement: Supplementary materials — Supplementary Table 1: qPCR analysis for Reg2, Reg3, Amy2b, and TLR4. MSC-Exo treatment increases Reg2, Reg3, and Amy2b expressions. Along with this, MSC and MSC-Exo treatment attenuated STZ-induced increase in the level of TLR4 mRNA. Data represent as the mean ± SD; ∗p < 0.05 versus the STZ+PBS group, by a two-tailed unpaired t-test. [file 9534574.f1.zip › Raw blot.jpg]
